# Microfluidics Technology for the Design and Formulation of Nanomedicines

**DOI:** 10.3390/nano11123440

**Published:** 2021-12-18

**Authors:** Eman Jaradat, Edward Weaver, Adam Meziane, Dimitrios A. Lamprou

**Affiliations:** 1 School of Pharmacy, Queen’s University Belfast, Belfast BT9 7BL, UK; ejaradat01@qub.ac.uk (E.J.); eweaver01@qub.ac.uk (E.W.); 2 Fluigent, 94270 Le Kremlin-Bicêtre, France; adam.meziane@fluigent.com

**Keywords:** drug delivery, liposomes, microfluidics, nanoparticles, nanomedicine, PLGA

## Abstract

In conventional drug administration, drug molecules cross multiple biological barriers, distribute randomly in the tissues, and can release insufficient concentrations at the desired pathological site. Controlling the delivery of the molecules can increase the concentration of the drug in the desired location, leading to improved efficacy, and reducing the unwanted effects of the molecules under investigation. Nanoparticles (NPs), have shown a distinctive potential in targeting drugs due to their unique properties, such as large surface area and quantum properties. A variety of NPs have been used over the years for the encapsulation of different drugs and biologics, acting as drug carriers, including lipid-based and polymeric NPs. Applying NP platforms in medicines significantly improves the disease diagnosis and therapy. Several conventional methods have been used for the manufacturing of drug loaded NPs, with conventional manufacturing methods having several limitations, leading to multiple drawbacks, including NPs with large particle size and broad size distribution (high polydispersity index), besides the unreproducible formulation and high batch-to-batch variability. Therefore, new methods such as microfluidics (MFs) need to be investigated more thoroughly. MFs, is a novel manufacturing method that uses microchannels to produce a size-controlled and monodispersed NP formulation. In this review, different formulation methods of polymeric and lipid-based NPs will be discussed, emphasizing the different manufacturing methods and their advantages and limitations and how microfluidics has the capacity to overcome these limitations and improve the role of NPs as an effective drug delivery system.

## 1. Introduction

Nanotechnologies are one of the most inspiring technologies in recent centuries that appear as a novel and promising research filed. It gained awareness and attention when the American physics Nobel laureate Richard P Feynman discussed the hypothesis about nanotechnology in his lecture “There’s plenty of room at the bottom” at a conference by the American Physical Society in December 1959 [1]. However, in reality, nanoparticles (NP) structures are not new; these structures have existed on the earth since ancient times; for example, Romans used NPs in glass manufacturing from the fourth century AD, since they used Nano-glass particles to fabricate a glass cup, acknowledged as Lycurgus cup, which was famous and distinctive due to its contrasting colour appearance under the different tones of light [2]. These days nanotechnology is a science in itself, with several applications in different fields, including water purification, information technologies, drug development, environmental, food industry, and making more robust and lighter materials [3,4].

The nanotechnology topic includes creating and manipulating nanometre-size materials by reducing bulk materials or scaling up atomic materials. NPs are small particles that vary in physical dimensions from 10 nm to 1000 nm. The engineered nanoparticles are commercial particles created especially with dimensions less than 100 nm [2].

NP structure is composed of specific molecules produced at the molecular and atomic level. NPs possess significant chemical, structural, electrical, biological, and mechanical characteristics. There exist diverse groups of NPs, including fullerenes, ceramic, polymeric, metal and lipid-based NPs. The value of these materials was recognized when researchers found that size can influence the physicochemical properties of the materials [5]. For example, the optical properties of gold and silver NPs depend mainly on the particle size, which conveys different colours because of the absorption in the visible spectrum [6]. In addition, toughness, melting point, reactivity, thermal conductivity, quantum effects, and other properties are related directly to a NP’s unique size and morphology. Moreover, Nanotechnology’s unique properties show a major improvement in the industrial area, with a great performance in biotechnology, aerospace engineering, and electronics [2].

Many researchers have realized a significant impact of NPs in improving various medical fields such as in the biomedical and pharmaceutical applications, as well as tissue engineering and in vivo imaging. NPs have a special extended medical importance in enhancing the drug delivery system, including encapsulating and targeting the delivery of molecules (e.g., chemotherapy) and biologics (e.g., DNA, proteins, and antibodies). Nanomedicines (NMs) have the potency to surmount the disadvantages and limitations of conventional drug administration, such as insufficient efficacy, poor biodistribution, lack of sensitivity, and toxicity. Therefore, nanotechnology represents an active area for research to improve drug formulations, controlled drug release and targeted delivery.

## 2. Nano Based Drug Delivery Systems

Applying nanotechnology in the medical field was an encouraging step, especially in improving the diagnosis and treatment methods. Many nanotechnology applications have been reported in medicine, including in vivo and in vitro diagnosis, drug delivery, nutraceuticals, and producing biocompatible materials [7,8]. Engineered NPs are an essential tool to achieve some of these applications. In the medical field, not all of the used particle sizes comply to the stated definition of nanoparticles with a size of ≤100 nm [9]. Hence, the particle size is not only what affects the functionality of NPs in medical applications; the main reason for using NPs is their essential characteristics, such as high mass-to-surface-area ratio, their ability to adsorb and function as a carrier to the other compounds, and their quantum properties. NPs possess a significantly large “functional” surface that can adsorb, bind, and carry different compounds, like proteins or other functional moieties. Depending on their chemical properties and morphology, NPs can be classified into several categories such as ceramic nanoparticles, carbon-based nanoparticles, semiconductor nanoparticles, metal nanoparticles, lipid-based nanoparticles, and polymeric nanoparticles [10,11]. The entire system drives to a particular purpose associated with treating, diagnosing, and preventing disease, and it’s termed smart-drugs or theranostics [12].

The main objective of nanotechnologies in drug delivery is increasing the specificity of drug targeting and delivery, improving safety and the biocompatibility, reducing the toxicity whilst maintaining the therapeutic effects simultaneously. The controlled drug delivery systems (DDSs) are based on carrying and transporting the active pharmaceutical ingredients (APIs) to reach the exact site of action. Consequently, it affects the essential and desired tissue and minimizes any unwanted effects. DDS can also keep the drug away from the accelerated degradation or clearance by increasing the drug concentration at the target tissue. Lower doses of the drug are therefore needed, potentially leading to reduced manufacturing costs [13]. It is essential to apply this modern therapy mode when the relationship is discrepant between the drug dose or concentration and the therapeutic effect or toxicity. Attaching the drug to individually created carriers can achieve cell-specific targeting and the recent advancements in nanotechnology record that NPs with dimensions less than 100 nm, are excellent as drug carriers [12]. Owing to the nanosize of these structures, they possess unique biological and physiological properties, such as the capacity to cross tissue and cell barriers, making them preferable for biomedical applications [14].

NPs also hold a higher possibility of being taken up by cells more than other particles due to their larger surface area, which permits in some cases increasing of protein loading by adsorption [15]. When NPs have any contact, especially with biological substances, it’s coated with various proteins called a “protein corona”. NP corona has a primary component named “opsonins”, which are directly responsible for enhancing the NP uptake by the “Reticuloendothelial system” RES [15,16]. Therefore, it can be used as a functional carrier for targeted drug delivery to the disease site in order to increase the poorly soluble drug uptake, target the drugs to a specific location, and enhance drug bioavailability [16].

## 3. Application of the Nano-Drug Delivery Systems

The application of NPs in nanomedicine, have been investigated in different areas, from controlled drug delivery to the diagnosis and prevention of diseases. Therefore, this section discusses NP potential for targeted biological molecules delivery (e.g., siRNA) for nucleic acid-based treatment and a nanoparticle’s potency in targeting chemotherapy delivery to different types of cancerous cells. Highlighted below in this section are few emerging applications for nanoparticles within the medical filed. The authors would like to note that nanoparticles have a broader scope than those mentioned in this article.

### 3.1. Nanoparticles for Nucleic Acid-Based Treatment

Nucleic acid-based treatment is one of the promising techniques for drug delivery targeting, especially to treat genetic disorders and cancer. For example, the plasmid [17] can be utilized to repair the defective genes, and the small interfering RNA (siRNA) [18] can be utilized to regulate the therapeutic process[19]. Compared to conventional therapeutic molecules, Nucleic acid-based treatments need delivery carriers to facilitate their entry into the cells and protect them from nucleases and other agents[19]. NPs possess many advantages over molecular medicine, giving them the potential to surmount various challenges and limitations of nucleic acid-based treatment, particularly the therapeutic agent’s bio distribution and bioavailability. NPs’ superior retention in vivo is an exceptional property through the decrease in enzymatic degradation and sequestration by phagocytes of the RES [20]. That property is related to their immunochemically inert surfaces in association with the biological environment. Via compromised vasculatures, NPs can guide the precipitation of the drug to the site of disease in a phenomenon called enhanced permeability and the retention effect also participates to their enhanced deposition in the disease areas [21]. siRNAs are used in various applications as a robust way to control gene expression. The free form of siRNA is unstable in the bloodstream and is unable to cross cell membranes and tissues. Different NP types have been used for delivering the RNAi, for example, Quantum dots (QD) have been used to control the delivery of RNAi [22], NPs based polylactic acid (PLA), and Poly(lactic-co-glycolic) (PLGA), have also applied for the delivery of RNAi in vitro [23]. Some success in delivering siRNA can be noticed using several nanomaterials (e.g., chitosan, PLGA, and lipid NP). Tracking their delivery and observing their transfection efficiency is complicated unless a proper tracking agent or tag is used. At the same time, there’s significant difficulty to produce an effective and self-tracking transfection agent for RNA interference.

Latterly, Tan et al. [24], designed a nano-carrier by synthesizing chitosan NPs and encapsulating them with quantum dot nanomaterials to transport the human epidermal growth factor receptor-2 (HER2/neu) siRNA [24]. It acts as a new and unique nano-carrier that helps in monitoring the delivery of siRNA due to the existence of fluorescent QDs into the encapsulated chitosan NPs. The targeted delivery of HER2 siRNA to reach HER2 receptors (an overexpressed receptor by SKBR3 breast cancer cells) can be more specific using chitosan—QDs technology. This nano-carrier can be functionalized by grafting the NP with HE ER2 antibody groups to enhance the target specificity [24].

Marking NPs with a fluorescent label, such as Cy-5, aids in visualizing the process of nanotube uptake and accumulation by using fluorescent microscopy. Howard et al. [25], integrated chitosan NPs with siRNA and utilized it mainly to the BCR/ABL-1 junction sequence. A 90% decrease is detected in the expression of BCR/ABL-1 leukaemia fusion protein in K562 (Ph (+)) cells. Also, using nasal administration of chitosan/siRNA formulations in vivo drives the RNA molecule effectively to inhibit the gene expression or translation in the bronchiolar epithelial cells of transgenic EGFP mice (RNA interference).

The same characteristics and features that have been approved in chemotherapeutic delivery are confirmed to be used in siRNA delivery and packaging [26,27]. The siRNA delivery and packaging is started by combining siRNA molecules with the NPs in a stable way and retaining them in circulation. Several techniques, such as complexation and the encapsulation and the non-covalent union of siRNA within numerous nano-constructs have been reported in combining siRNAs to different biocompatible and inert molecules, including long-chain fatty acids and cholesterol [28,29]. Unfortunately, the success is still restricted until this day, due to the different challenges and limitations faced during the delivery process. Moreover, recently issued reports discussed the toxicity and instability of many siRNA-nanoparticle complexes in vivo [30,31].

### 3.2. Nanoparticles for Cancer Cell Targeting

Today, cancer exhibits as a significant hurdle in the area of pathology. The lack of chemotherapy selectivity and the random attack on the healthy cells is one of the main limitations that directly affects the treatment’s safety and efficacy. It also produces severe side effects, and can generate insufficient drug concentrations within the desired tumour tissues. Applying NPs for therapeutic purposes, particularly for targeting the delivery as DDS, makes a significant development in the drug administration systems and a potential role in overcoming the limitations of chemotherapy.

One of the NP’s recent applications is the use of glyconanoparticles targeting prostate cancer cells. The glyconanoparticle is a highly branched polymer of glucose, functionalized with lactose to add the β-galactosidase side to it and named as galacto-glycogen (GG) [32]. GG, can fully interact with lectins, such as the Galectin-1 (Gal-1) and peanut agglutinin (PNA) [32]. Gal-1, is a β-galactosidase binding protein highly expressed on the PC3 prostate cancer cells and plays a vital role in the progression of the disease by making multiple interactions with endothelial and other cells [33]. Various manufactured platforms have been employed to enhance the attraction between the glycoconjugates and their receptors, for example, glycopolymers [33], glycopeptides [34], sugar-functionalized gold [35], glycodendrimers [36], iron oxide NPs [37], silica, liposomes [38], and carbon nanotubes [39]. The engineered GG shows a high affinity to the cancer cell membrane and a strong interaction with Gal-1, which makes them capable of inhibiting the Gal-1 proliferative activity or as being used as a carrier to target the delivery of an anticancer drug to the PC3 cells [40].

Other applications of using NPs in cancer therapy have also been reported. There’s serious difficulty in passing chemotherapeutic agents through the blood-brain barrier (BBB) and towards the brain [41,42]. Researchers have discovered that NPs hold a potency for carrying therapeutic active ingredients into the brain. Apolipoprotein E is a recommended receptor for stimulating drug transportation across the BBB [43]. For example, Loperamide is a drug with an antinociceptive effect but lacks the capacity to pass the BBB; therefore, when it’s administered directly to the brain by intracerebroventricular injection, it exerts antinociceptive effects. The drug was loaded into human serum albumin NPs and attached to Apolipoprotein, then administrated intravenously to mice; the results confirmed an antinociceptive effect in the tail-flick test. Typically, the efficiency of that complex drug delivery system relies on lipoprotein receptor recognition.

Doxorubicin (another form of chemotherapy) can access the brain and come across the intact BBB and release the therapeutic concentrations when it’s bound to polysorbate-coated NPs [44]. Various anti-cancer drugs, counting doxorubicin-5-fluorouracil, dexamethasone, and paclitaxel, are formulated conventionally by nanomaterials. For example, PLGA (polylactic co glycolic acid) and PLA (polylactic acid) based NPs are used to encapsulate dexamethasone (a glucocorticoid that acts intracellularly at the site of action) [45].

An additional application of a special nanoscale sensor can be used effectively to target cancer cells. The Probes Encapsulated by Biologically Localized Embedding (PEBBLE), is a nanoscale sensor that Kopelman designed with potency for carrying diverse kinds of individual molecules on their surface and displays many functions [42]. Fixation of a target molecule on the PEBBLE surface permits its distribution directly to the tumour site. Also, a second agent could be attached to help in visualizing the target in magnetic resonance imaging (MRI). In contrast, another agent can be joined to PEBBLE to cooperate in the delivery of fatal dose of drug or toxin adjacent to the cancer cells. These agents’ capacities can be merged in a small individual polymer sphere to create powerful weaponry against cancer. Therefore, different platforms of nanomaterials perform as an optimum drug carrier to target cancer cells.

### 3.3. Nanoparticles and Angiogenesis

The circulatory system plays a fundamental role in supplying the cells with oxygen and nutrients to maintain a cell’s growth and development [46]. Cancerous cells, like healthy cells, need blood circulation for sustained feeding with oxygen and nutrients to speed their growth and invasiveness. The tumour cells induce vascularization (angiogenesis) to receive their needs and enhance tumour metastasis [47].

Angiogenesis means the generation of new blood vessels from the existing vessels after the tumour cells release angiogenic factors such as vascular endothelial growth factor (VEGF) [47]. Due to the fundamental role of angiogenesis in tumour cell survival, suppressing angiogenesis’s formation is the primary mechanism to repress tumour progression. Angiogenesis can be suppressed by inhibiting the angiogenesis stimulating factor such as integrin’s [48]. Integrins are a group of proteins that represent a critical part in tumour migration, metastasis, and survival; these integrins’ tumour cell expression is linked to diseases in several kinds of tumours such as breast cancer, prostate cancer, pancreatic cancer, and melanoma [49]. At first, the only integrins targeted to repress tumour angiogenesis were αvβ3 and αvβ5, but now researchers have targeted a vast collection of integrin’s such as α1β1, α2β1, α3β1, α4β1, α5β1, α6β1, αvβ5, αvβ6 and αvβ3 to repress tumour angiogenesis [50]. Recent reported studies notes that αvβ3 integrin and vascular endothelial growth factors (VEGFs) perform the primary regulatory functions for angiogenesis [51]. Hence, selective targeting of αvβ3 integrin and VEGFs is an innovative method to treat various solid tumours. One of the approaches is to use NPs coated with peptides that specifically bind to the αvβ3 integrin and the VEGF receptor [51]. The peptide-coated NPs have an ArgGly-Asp (RGD) sequence, which binds especially to the αvβ3 integrin presented on endothelial cells in the angiogenic blood vessels and probably inhibits tumour extension and proliferation. A reported study proves that using αv binding agents to targeted chemotherapy is a useful technique, which applied an ArgGly-Asp (RGD) peptide joined to doxorubicin to enhance this chemotherapeutic drug uptake. Doxorubicin effectiveness has been experimented with in breast cancer xenografts in nude mice [52]. NP-mediated αvβ3 targeting agents that contain different therapeutics have been examined [53].

Murphy and co-workers performed another study about doxorubicin delivery [54] by synthesizing a αvβ3 targeting NP, capable of delivering doxorubicin into the tumour vasculature. These NPs consist of distearoylphosphatidylcholine (DSPC), cholesterol dioleoylphosphatidylethanolamine (DOPE), and distearoylphosphatidylethanolamine (DSPE)-mPEG2000 simultaneously, alongside the RGD and was successful in incorporating doxorubicin. The same authors have also reported in a different study that these NPs have the ability in controlling pancreatic cancer and renal cell carcinoma metastasis in orthotopic mouse models [54]. The experiments showed a 15-fold enhancement in the dose-response of the drug to the tumour vascularization when using the encapsulated doxorubicin in RGD guided NPs instead of using free doxorubicin [49].

Other studies have been investigating tetraiodothyroacetic acid (tetrac), a thyroid hormone analogue [55]. The anti-angiogenic qualities of tetrac have been confirmed to be relevant to the vascular supply of tumours alongside the capability to attack a plasma membrane receptor on integrin αvβ3 [55,56]. Due to all the powers of tetrac, laboratories have started working on developing a nanoformulation integrating the biodegradable polymer poly(lactide-co-glycolide) (PLGA) with tetrac [55,56,57]. However, the free form of tetrac can easily penetrate the cell nucleus and exhibit adverse genomic effects (when administered as an anticancer agent). The nanoformulation strategy in combining tetrac to PLGA nanoparticles (T-PLGA-NPs) can resolve this complication by stopping NPs from accessing the nucleus. The NPs larger size is the principal reason for preventing them from going into the nucleus [58]. Simultaneously, this property of the NPs allows the utilization of this nanoformulation as a therapeutic means to study the anti-angiogenic and anti-proliferative effects of tetrac via the integrin’s extracellular domain [58].

### 3.4. Nano Systems in Inflammation

The power of macrophages in the rapid detection and clearance of foreign substances provides a rational strategy for controlling inflammation using macrophage-specific targeting with NPs. The ability of Macrophages to secrete several inflammatory mediators enables them to control inflammation in various conditions; for example, activation signals comprise cytokines (e.g., interferon-gamma, colony-stimulating factor, tumour necrosis factor-alpha, and granulocyte-monocyte), extracellular matrix proteins, and bacterial lipopolysaccharide. Hence, they have a primary role in muscle repair, wound healing, and cancer. Macrophages can kill most microbes and many microorganisms such as Leishmania [59], Listeria monocytogenes [60], and Mycobacterium tuberculosis [61].

NPs that mediate antimicrobial agent(s) delivery inside pathogen-containing intracellular vacuoles in macrophages could help in excreting cellular reservoirs [62]. By this method, greater than one purpose can be reached. It can help achieve the therapeutic drug concentrations in the vacuoles of infected macrophages whilst reducing the unwanted effects correlated to drug administration and free the pro-inflammatory cytokines. Another example of nanoformulations used as carriers into macrophages is the Polyalkylcyanoacrylates (PACA) NPs, which are used for targeting the anti-leishmanial drugs. This nanomaterial inhibits the macrophages’ ability to release interleukin-1 [21]. Hence, nanosystems designed in a similar way could be extremely beneficial in targeting macrophage infections in chronic diseases.

Amphotericin B (AmB), an antifungal and anti-leishmanial therapy, has been combined with lipid-based nanotubes for investigating the generation a less toxic formulation of AmB. Gupta and Viyas attempted to formulate AmB in trilaurin-based nanosized lipid particles (emulsomes), then stabilised the complex with soya phosphatidylcholine (PC) to produce a novel system for the intravenous (IV) drug delivery for macrophage targeting. Macrophage’s nanocarrier-mediated delivery for toxins has been confirmed as a potent strategy to clear out the undesired macrophages in gene therapy and other associated clinical conditions, including rheumatoid arthritis, autoimmune blood diseases, T cell-mediated autoimmune diabetes, sciatic nerve injury, restenosis after angioplasty, and spinal cord injury [63]. On the other hand, the lethal properties of NPs with macrophages can be utilized, and demonstrate a more useful antigen delivery approach and target a particular nanocarrier [64].

However, the potential success of NPs platforms in the clinical trials as drug carriers relies on essential parameters such as NP fabrication strategies, their physical properties, drug loading efficiencies, drug release potential, and most importantly the toxicity of the carrier itself. Among these platforms, lipid-based nanoparticles have some of the lowest toxicity in in vivo experiments, having the ability to carry hydrophilic or hydrophobic molecules and extend the duration of action, which means prolonged half-life and a controlled drug release. Lipid-based NPs can be subjected to chemical adjustment to bypass the immune system’s detection, such as PEG, or can be attached to antibodies to recognize the tumour cells receptors, like folic acid (FoA) [65]. Lipid NPs can be prepared as sensitive PH formulations to enhance the drug release in acidic conditions [66]. All of that, promotes the efforts to expand the studies on lipid NPs development, scale-up production, and manufacturing methods.

## 4. Manufacturing Methods for Lipid Formulations

Lipid-based NPs formulations, including liposomes and solid lipid nanoparticles (SLN), have been manufactured using different methods. The several manufacturing methods can be categorized into conventional methods and novel methods. The varying use of manufacturing method mainly impacts the final quality of the NPs with their size, polydispersity index (PDI), and morphology. Different lipid-based NPs manufacturing methods are discussed in this section.

### 4.1. Liposomes

Based on years of research, liposomes have achieved importance and interest in biological, pharmaceutical, and medical research, as the most effective carrier for a variety of molecules in cell targeting and other various applications. For example, Doxil^®^ is a form of liposomal chemotherapy used to treat different cancer cells (e.g., Breast cancer, bladder cancer, and lymphoma). A liposome, is a microsphere lipid constructed from one or multiple phospholipid bilayers, closely mirroring the cell membranes’ structure. Liposomes have a broad spectrum of applications extending from employment in basic research in biophysics to various practical applications like cosmetics, pharmaceuticals, production of ultrafine particles [67,68].

According to the definition that issued by the New York Academy of Sciences [64], liposomes are classified to three categories: multilamellar large vesicles (MLV; 0.1–6 μm), small unilamellar vesicles (SUV; 0.02–0.05 μm), and large unilamellar vesicles (LUV; >0.06 μm). Other shapes (than spherical) and structures have been reported, including oligolamellar [69], giant unilamellar, multivesicular [67], stable pausilamellar [68], helical [69] and cochleate [70]. The liposome preparation procedure can be generalized and split into three stages [70]:

1. Preparation of the aqueous and lipid phases;
2. Primary processing involving lipid;
3. Secondary processing steps (essential in some formulations and optional in others);

Practically, all methods require the dissolution of phospholipids in an organic solvent; after that for most methods, the organic solvent should be removed by the evaporation during the process. The previous dissolution, followed by removing the organic solvent, is a critical step in the liposome’s formation process.

Phospholipids and cholesterol are the main building blocks of liposomes. The most commonly used phospholipids have a critical micelle concentration in the nanomolar scale. But phospholipids concentrations that are utilized in the liposome formation process should be higher than the critical micelle concentration. The three-dimensional cylinder shape of phospholipids stimulates liposome formation, that when phospholipids are exposed to the aqueous atmosphere, the lipids start to assemble and aggregate. Mixing the lipid and the aqueous phase can be achieved by in a few ways such as creating a thin lipid layer before exposing it to the aqueous phase or inserting the lipid phase in the aqueous phase in a controlled flow rate to form liposomes. Due to this, all of the liposome formation methods, such as solvent dispersion/antisolvent, solvent evaporation (lipid hydration), or detergent removal, concentrate mainly on breaking phospholipids into single phospholipid molecules [71]. Then, exposing it to an aqueous condition to allow forming several classes of liposomes, viz. MLVs, SUVs, GUVs, OLVs, MVVs [72].

The lately recorded techniques are classified as conventional, which often involves easy and simple methods for using on the laboratory scale; moreover, the novel techniques seem more useful for scaling up production due to using exceptional tools controlling the process [73].

#### 4.1.1. Conventional Methods

The common strategies to create liposomes followed the identical basic stages are:

- Lipids dissolution in organic solvents;
- Drying the obtained solution;
- Hydration of the dried lipid (applying different aqueous solution);
- Separation of the liposomal vesicles;
- Quality control assays.

##### Film Hydration

**Figure 1:**
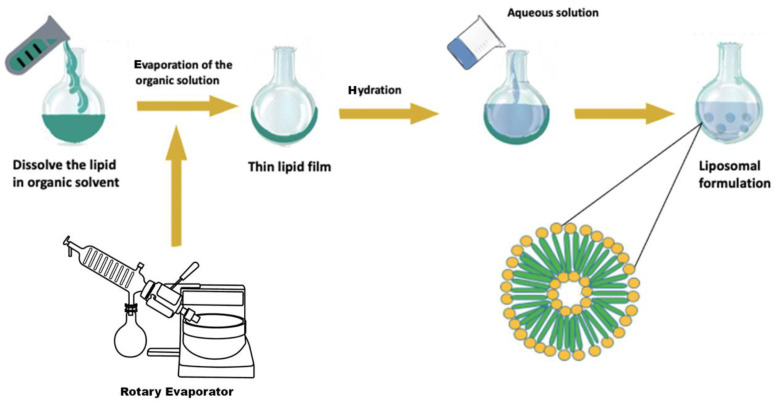
Thin film hydration method for empty liposome preparation. The liposomes produced from this method are often polydisperse; however, there is a correlation between the duration spent during the rotary evaporation step and the quality of thin film produced. Factors such as mixing speed and temperature after the hydration will also effect the quality of the liposomes so this must be monitored.

Film hydration resembles the most straightforward and the earliest approach applied in liposome science; it is named as the Bangham method. Firstly, the lipid is dissolved in an organic phase (e.g., Chloroform, methanol, dichloromethane or trichloromethane) then dried using a vacuum rotary evaporator it until a thin lipid layer is created in the bottom of the flask. After that, the resultant lipid film needs to be hydrated with a suitable aqueous fluid (e.g., deionized water) to provide dispersed liposomes (Figure 1). Hydration conditions can directly affect the structural organization of the obtained liposomes. Therefore, the soft and mild hydration of the lipid yields giant unilamellar vesicles (GULV). On the other hand, confusion hydration can increase the generation of MLV with inadequate size uniformity. The sizing methods commonly used are probe and bath sonication that allow the creation of SUV. Probe sonication has higher effectiveness compared to other methods. Still, at the same time, it is often condemned with the possible contamination (by titanium from the titanium-based nozzle, which is utilized for mechanical agitation), as well as the heat generated during the process affecting the stability of the lipid and drug. Even though both of the sonication strategies provide liposomes with similar properties, using the bath sonication method is still a more desirable choice because of the simple control of operational parameters. After the sonication the solution must be left to rest before being subjected to centrifugation. The main limitations of this method are the application of organic solvent and mechanical agitation, which produce large and uncontrolled particles sizes (this demands an additional downsizing step like the sonication), heterogeneous distribution of the particles size, poor encapsulation efficiencies of hydrophilic materials, scaling up difficulties, time-consuming and sterilization issues [73,74].

##### Solvent Injection

**Figure 2:**
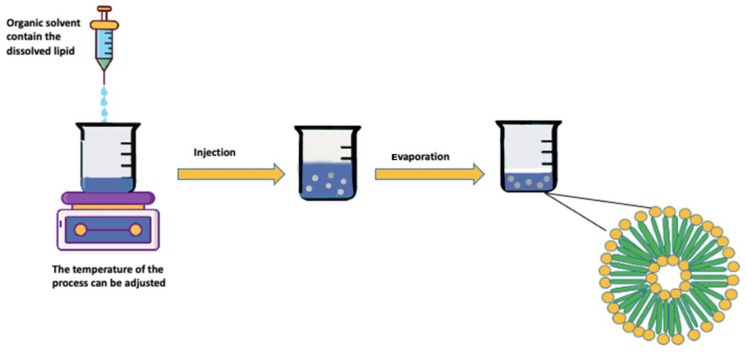
An illustration for the procedure of the solvent injection method to produce empty liposomes. The major factors to consider during the solvent injection method are the temperature during injection (as this will vary depending upon the phase transition temperatures of individual lipids), and the injection rate. These factors will affect the size, shape and polydispersity of the liposomes produced. A following method such as micro-extrusion is normally required after solvent injection to obtain a therapeutically viable formulation.

In solvent injection, the lipid solution will be injected at a fast-rate (ethanol or diethyl ether) into the aqueous phase (Figure 2). Room temperature or higher, like 60 °C, can be used to achieve this operation; this depends specifically on the level of organic solvent miscibility with water. Moreover, other parameters may affect the injection pressure and the ethanol to lipid ratio. Most of the time, solvent injection liposomes’ hold a relatively high polydispersity with distinguished organic solvent contamination, particularly ethanol, due to creating an azeotrope combination with water. The several drawbacks are the main problem that the solvent injection method has faced, mainly because the therapeutic substance is continuously exposed to the organic solvent at high temperatures, both of the conditions affect the liposomal products’ stability and safety [75,76]. The high polydispersity of the obtained liposomes will, as a consequence, indicate a non-homogenous formulation [77].

##### Detergent Removal

In this method, the primary step is preparing an aqueous solution containing a critical micelle concentration (CMC) of detergent, then dissolving the phospholipid within the solution. Through the process, the reaction’s medium causes the release of a single phospholipid, which begins to assemble around each counterpart, building bilayer structures. This process’s success depends on using polystyrene-based absorber beads or Sephadex columns (gel permeation chromatography) and a dialysis bag. The obtained compound should be diluted with an appropriate aqueous liquid; this can help reform the developed micelles, which contain the liposomes [73,74]. The weakness of this method, are the exposure to an organic solvent, time-consuming, detergent residual, inadequate yield, sterilization needed, and low entrapment performance.

#### 4.1.2. Re-Sizing of Lipid Suspension

Liposomes produced by conventional methods may hold large particle sizes or high polydispersity. The provided lipid suspension can be downsized using sonication. Sonication is a simple technique that breaks the aggregated large MLVs liposomes into small liposomes with low polydispersity. The sonication technique can be used for liposome preparation or as a second step of the liposome’s conventional manufacturing methods, as discussed in the film hydration method.

##### Sonication

Sonic energy, or in other words sonication, is a powerful energy that has the potential to alter a LMV suspension to SUV with a size range between 15 and 50 nm [78]. The sonication process’s accomplishment depends on the sonicators used, bath or probe tip. Probe tip sonicators, can pass a high amount of energy through the lipid suspension, but exposure the suspension to high-temperature heating can cause degradation. Also, the sonication tips can contaminate the suspension via releasing titanium particles, which need to be cleared by centrifugation. All these limitations of probe sonication were the reason to consider bath sonication as the most commonly used instrument for SUV preparation. The LMV dispersion is subjected to the sonication process by moving the container that contains the lipid suspension to the sonicator bath or to put the sonicator tip in the container itself, and sonicate the suspension for 5–10 min at a temperature higher than the Tc of the lipid. The suspension starts to refine into a lightly misty transparent solution. The mistiness of the solution is produced because of the light scattering of the large remaining particles in the suspension. The SUV solution becomes clear after removing these particles by centrifugation. The resultant particles’ diameter and polydispersity are affected by different factors such as sonicator tuning, the strength and duration of sonication, composition and concentration, volume, and temperature. The particle size variation in the produced batches at various times is expected, which is due to the difficulty of reproduction the sonication conditions. Because of the high-curving degree of the liposome membranes, the obtained SUV naturally shows a lack of stability, so it assembles impulsively to create more giant blisters if stored under their phase transition temperature [79].

The complexity of the liposome production offers many barriers such as the variability and distribution of particle diameter and “shell” deterioration. This is clearly displayed in the start-up difficulties, long development process, and bioequivalence studies that are compulsory when any modification occurs to the process. Thus, that makes the production of liposomes and art more than an area of study. Moreover, liposomes have an essential advantage of carrying the API in a targeted way to the active site. The targeting accuracy of the “homing” surface of the phospholipid/liposome can be easily created and designed for various applications. In general, liposome formulation still has physical and chemical stability difficulties, which might induce liposome assemblage and drug degradation during the storage period and compromise the administration of liposome-based drug carriers [79].

##### Extrusion

**Figure 3:**
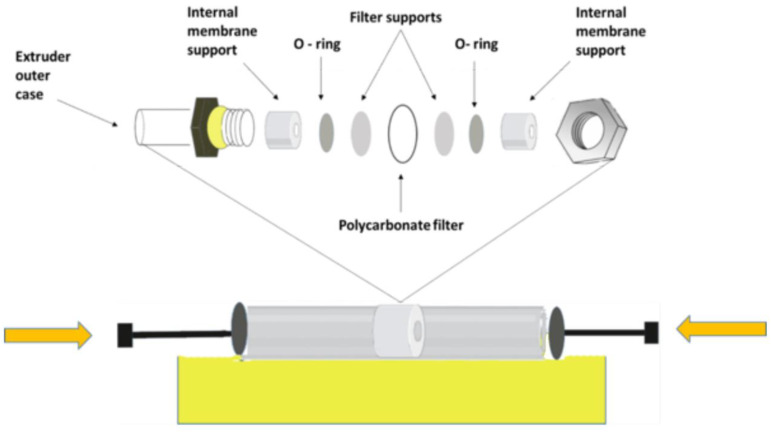
The preparation of liposomes by extrusion method using polycarbonate filters. Increasing the number of transitions through the membrane will reduce the polydispersity of the formulation. The process should also be performed at a temperature similar to that of the lipid transition temperature, to prevent lipid cleavage (and subsequent liposome breakdown) upon extrusion.

The lipid extrusion method, is driving a specific lipid-containing liquid through a filter pore size membrane producing particles precisely similar to the filter pore size. The lipid suspension is subjected to several passes through the polycarbonate filter (Figure 3). These passes can be forced by a machine with a pump (extruder) or can be manually in small-scale production. After these passes, the LMV suspension transforms into more petite and uniform SUVs or large LUVs. Between all the methods for reducing MLV size, the extrusion method needs to be at a temperature higher than the Tc of the lipid; all attempts to extrude below the Tc could be failed as the membrane become rigid and lose the ability to pass through the pores. Tc relates to the transition temperature of a material, where a material begins to transition between physical states. Various parameters can affect the diameter and polydispersity of the obtained liposomes using the extrusion method, especially the pore size. Extrusion through 100 nm pore filters produce LUV with a mean diameter of 120–140 nm [80]. Also, the applied pressure, number of cycles and the lipid composition could impact the process. This method has a central role in decreasing the polydispersity to produce a more homogenous final suspension liposome. On the other hand, the membrane extrusion technique has some problems in large-scale production, such as the clogging of the membrane pores, especially when the suspension is highly concentrated or when the diameter of the liposomes is much bigger than the pores. In addition, the pore surface area is only 20% of the whole surface area of the membrane that limits the available space for extrusion, consequently limits the extrusion output [79].

### 4.2. Solid Lipid Nanoparticles

Solid nanoparticles (SLN) have gained much attention in the last years as an alternative for other lipid formulation NPs such liposomes or polymeric NPs. SLN are colloidal particles with a spherical shape that vary in size from 10 to 1000 nm. The drug mobility in a solid lipid is more limited than other lipid formulations, which enhances the achievement of controlled drug release [81]. It has been realized that SLNs could hold the benefits and avoid the limitations of other colloidal carriers; for example, SLNs offer targeted the drug delivery and achieve controlled drug release [82]. Also, it has an increased stability, large capacity for scaled-up production, and bypasses the usage of organic solvents [81]. SLN are composed of a solid lipid core in an aqueous medium with a surfactant (emulsifier) [83]. The surfactant type is changeable due to the different route of administration as it is limited to use with the parenteral formulation. The solid lipid core consists of lipids such as steroids (cholesterol), fatty acid (palmitic acid, stearic acid), triglycerides (tripalmitin, trimyristin), complex fat types (witepsol), glyceryl monostearate (imwitor), and waxes (e.g., cetyl palmitate, beeswax) that possess a melting point higher than body temperature [84]. Stabilizing the lipid core-shell inside the aqueous phase requires adding surfactants or emulsifiers such as nonionic surfactants (pluronic, poloxamer), polyvinyl alcohol, polyethylene glycol (peg), ionic surfactants (sodium cholate, sodium lauryl sulfate), and phospholipids (soy lecithin, egg lecithin) [85,86]. The drug should be inserted into the gaps of the solid lipid matrix since the solid lipid core should be melted through the process then subjected to recrystallization at room temperature [83]. The drug insertion process relies on drug hydrophobicity, solid lipid category, and the polymeric alteration in the lipid crystals [87]. There are various techniques to produce and manufacture SLNs, including high-pressure homogenization, solvent evaporation, ultrasonication, hot homogenization, microemulsion, solvent emulsification–diffusion, double emulsion, microfluidics and supercritical fluid technique [88].

#### 4.2.1. High Pressure Homogenization Method

High-pressure homogenization (HPH) is an initial, reliable, and conventional method used for SLN manufacturing. The use of a homogenizer device is the central concept of this method; the liquids are propelled with high pressure (100–2000 bar) in a narrow gap (sub-micron dimension), and the fluid is subjected to a high velocity (above 1000 km/h) in a small area which generates high-stress to cause a downsizing of the particles to the submicron diameter [89]. HPH can be completed in two different conditions: hot pressure homogenization and cold pressure homogenization [90].

##### Hot Homogenization Method

The hot homogenization approach includes the preparation of SLNs at a temperature higher than the melting point of the used lipid; considered as emulsion homogenization. A pre-emulsion should have been prepared by mixing the appropriate lipid and drug then warming the lipid/drug mixture to a specific temperature above the melting point until melted, and on the other side, the aqueous phase with the surfactant is heated to the same temperature [91]. The aqueous phase is added to the drug-loaded lipid and the mixture is equilibrated to the same temperature. The quality of the pre-emulsion can highly affect the end product quality as it’s a desire to have droplets within the micrometer size. The pre-emulsion is subjected to external mixing power to disperse the emulsion at different speeds graduated from 5000 to 30,000 rpm; the varying rates of mixing is practiced with different durations times [91]. Finally, the lipid should be cooled to obtain the SLN; the cooling conditions could be gradually at room temperature or accelerate the process by using a refrigerator [91]. Also, it may be cooled by using cold water during the mixing at low speed (5000 rpm) or by adding the lipid to cold water with a considered ratio such as 1:10 emulsion to water (water temperature between 2 and 25 °C) [91]. The speed of the mixing, the duration of emulsification, and the cooling states, are all essential parameters that determine the final particle size and ζ-potential. To achieve high-quality SLN production, it must be consider that the higher preparation temperature obtains a smaller particle size due to the lower viscosity of the lipid solution [92]. Also, multiple passes through the high-pressure homogenization can increase the emulsion temperature (about 10° at 500 bar), generally 3 to 5 passes (at 500–1500 bar) are enough to have the best results; if the number of passes increases, the particle size will be increased and coalescence may occur [93]. Like any other methods, this method has also some limitations, such as the high consumption of mechanical energy, the lack of control on the effective particles size, long term stability, and the loading capacity, as well as the high cost of production due to the multiple steps of preparation and the high energy requirement [94]. All these restrictions boost the researchers to investigate other novel methods to overcome these limitations.

##### Cold Homogenization Method

The second version of the high-pressure homogenization is the cold homogenization method, which is regarded as the milling of a suspension. The entire process of this method needs to be under a well-controlled temperature to keep the material in a solid-state [93]. The cold homogenization method is developed to overcome the limitations of the hot homogenization method, such as the complication of the nanoemulsion crystallization process that may cause some modification and supercooled melts, the potential drug degradation due to the high exposure to heat, and the possible splitting up and hence loss of the drug into the aqueous phase during the homogenization process [89].

The first step of the cold homogenization method is comparable to the hot homogenization, as the desired drug is dispersed into the melted lipid. Then, the drug-loaded lipid is cooled using liquid nitrogen or dry ice. The cooling process should be completed quickly to have homogenous drug distribution in the lipid matrix. Then the active solid lipid is subjected to milling using a ball or mortar miller to produce microparticles in the range of 50–100 microns. Chilled processing further promoted particle milling by increasing lipid fragility [89]. Disperse the solid microparticles into a surfactant solution. The pre-suspension undergoes high-pressure homogenization at or below room temperature with proper temperature. The high-pressure process usually raises the temperature spontaneously, so concise monitoring of the temperature change is needed [95].

By comparing the cold homogenization with the hot homogenization samples, the cold homogenization produces solid NPs with larger dimensions and less homogeneity (high PDI) [96]. However, the cold homogenization method’s advantages include the reduction of exposure to heat; drug-loaded lipids are exposed to the heat only in the first step of the process.

#### 4.2.2. Ultrasonication or High Speed Homogenization

High-speed homogenization or sonication is one of the most straightforward dispersing techniques to produce SLNs [97]. The drug and the suitable Phospholipid are mixed and added to an aqueous solution to prepare a pre-emulsion. Then, the pre-emulsion is subjected to high-speed homogenizing for a sufficient period of time [98]. During the process, it is recommended to keep the temperature 5 °C above the lipid melting point and ultrasonicate the pre-emulsion for 15 min after the mixing to avoid lipid crystallization [99]. The oil/water (o/w) emulsion should be cooled at room temperature with continuous stirring to recrystallize the lipid and SLN created [99]. The main limitation of this method is the high polydispersity of the produced particles, which affects the physical stability of the particle directly and induces particle growth in storage, alongside the possible metal contamination [89]. Several research groups have combined high-speed stirring and ultrasonication and perform the method at high temperatures in order to increase the formula’s stability and overcome the limitations [81].

#### 4.2.3. Microemulsion

A lipid-based microemulsion is prepared by the continuous stirring of an optically transparent solution constituted of emulsifier (e.g., polysorbate 20), low melting temperature lipid (e.g., stearic acid), and water at a temperature close to 65–70°. The obtained microemulsion should be dispersed with continuous stirring in cold water (2–3 °C) [100]. The volume ratio of the water medium and microemulsion are in the range of 25:1 to 50:1, and the dilution procedure is decided according to the composition of the microemulsion [101].

The highlighted advantage of this method is that the microemulsion contains the droplet structure spontaneously without the need of using external energy to get the submicron size. According to the literature [102], the particle size of SLNs is determined mainly by the speed of the distribution process; the hydrophilicity of the used solvent can impact the size of the SLN. Using a hydrophilic solvent can enhance the distribution velocity in the aqueous phase compared to the nanoparticles that are distributed in hydrophobic solvent, which develops larger particles [81]. Considering microemulsions, the construction of the microemulsion as well as the temperature gradient forms the product quality. High-temperature gradients promote rapid lipid crystallization and avoid aggregation [81]. The main limitation of this method is the low concentration of the produced NPs, alongside the need for a high quantity of water and the high sensitivity to change [103].

## 5. Manufacturing Methods of Polymeric Formulations

Polymeric nanotechnology has a primary role in current investigations in DDSs, due to its specified chemical composition, polyvalent surface, and three-dimensional construction [104]. Both natural and synthetic polymers are developed into special nanocarriers to enhance drug loading capability, target the action site, and control drug release [105]. Different formulations of NPs can be formulated using polymers, including nanocapsules, nanospheres, and emulsion droplets. The various types of polymers that are used for the manufacturing of NP carriers are approved materials by the Food and Drug Administration (FDA), and includes hydrophobic polymers such as polycaprolactone (PCL), poly-lactic acid (PLA), poly-(lactic-co-glycolic acid) (PLGA), and hydrophilic polymers such as albumin, chitosan, gelatine, and alginate [104]. Natural polymers are biodegradable and biocompatible, confirming a complete elimination from the body and making them less toxic than synthetic polymers [106]. However, the use of natural polymers is still limited due to poor batch-to-batch reproducibility and the possible immunogenicity [107]. The sustained release of the incorporated drug over days to weeks is the main advantage for synthetic hydrophobic polymers over natural hydrophilic polymers. However, the use of synthetic polymers is still limited due to the high organic solvent usage and the possible toxicity [105].

Recently, many efforts have been centred on preparing well-characterized NPs by choosing the most suitable polymer type, preparation method, and other factors that can highly affect the quality and performance of the polymeric nanoparticles in vivo [107]. Different methods are used to manufacture polymeric NPs, which can be categorized into two groups: two-step processes that need to prepare the emulsification system as the first step and then form NPS, and the one-step process that exempts the emulsification step. A comparison between the different manufacturing method of polymeric NP and lipid NP are in (Table 1).

### 5.1. Two-Step Emulsification Procedures

In two-step procedure, the first step is emulsifying the organic polymer solvent (including the drug) and the aqueous solution. An emulsifier device should be used to produce the nanodroplets then the NPs [114]. In these methods, the droplet formation step is the control step that determines the obtained polymeric nanoparticle (PNP) size and homogeneity [107]. After the nanodroplets are formed, the organic solvent should be eliminated to condense the polymers into the nanodroplets and form the PNP [107]. Several methods are used for removing the polymer organic solvent, such as fast diffusion after dilution, solvent evaporation, or salting out. In all the method procedures, the drug is added to the polymeric organic solvent as the first step to encapsulate it [107].

#### 5.1.1. Emulsification-Salting Out

The salting-out method is a simple method based on emulsification. The emulsion is composed of a water-miscible solvent (e.g., acetone) containing the drug and an aqueous gel including a surfactant and salting-out agent. Electrolytes are examples of salting-out agents, including calcium chloride, magnesium chloride or non-electrolytic salting agent such as sucrose [107]. The emulsification process is accomplished without using homogenization energy, as the ouzo effect is the causative of the homogenization process [115]. The miscibility of water and acetone is reduced, and an o/w emulsion is formed by increasing the saturation of the aqueous phase. The obtained o/w emulsion should be diluted with a high amount of water to induce the diffusion of the acetone into the aqueous phase and get a reverse salting-out effect. The reverse salting-out effect will facilitate the precipitation of the dissolved polymer in the nanodroplets. The remaining salting-out agent and polymer-solvent can be removed using cross-flow filtration [116].

This method holds an advantage avoiding the use of chlorinated solvents, which harm the physiological system and environment. The main disadvantages are the limited utilization for the lipophilic drugs encapsulating and the need for additional purification steps due to the using salts [107].

#### 5.1.2. Emulsification-Solvent Diffusion

The solvent diffusion method is an advanced method of the salting out method [107]. The first step of the emulsification-solvent diffusion method is preparing an emulsion that includes a partially water-miscible solvent with the polymers and drug, and an aqueous solution with surfactant. To succeed in this process, the polymeric solvent solution and aqueous solution should be mutually saturated at room temperature to ensure the initial thermodynamic equilibrium of the liquids [107]. After the droplets are formed, the mixture is diluted with a high quantity of water and the colloidal particles formed [117]. In general, this method can obtain nanospheres, but adding a small amount of oil to the organic solvent is required to get nanocapsules. This method shows clear advantages such as the scaling up probability, high yields, the needless of high-pressure homogenizer, the high encapsulation capability, batch-to-batch reproducibility [118]. The disadvantages of this method include the increased water consumption, the potential loss of the water-soluble drugs in the external phase during the emulsification process [119], low stability of the emulsion, and the low solubility of the lipids in organic solvent [103].

#### 5.1.3. Emulsification-Solvent Evaporation

Emulsification solvent preparation is the first applied method to prepare PNP with the desired properties [107]. This method, as a simple and versatile method, was applied successfully to produce drug carriers using biocompatible polymers. The hydrophobic drug and polymer are dissolved in a suitably volatile solvent as the first step. Previously, chloroform and Dichloromethane were used as a solvent; however, they are now substituted with ethyl acetate, which has a safer toxicological profile and greater compatibility with biomedical applications [118]. The resultant organic solution is subjected to emulsification in an aqueous solution utilizing a surfactant and high-speed homogenization to produce a nanodroplet dispersion [120]. The evaporation of the polymer-solvent is the critical step to obtain a NP suspension. The polymer-solvent is enabled to diffuse through the continuous emulsion phase and can be evaporated by constant magnetic stirring at room temperature or by being subjected to reduced pressure, which is a slow process [121]. The produced PNP can be collected by centrifugation then put through freeze-drying for long storage purposes [122].

The solvent evaporation method is simplistic and versatile. The main limitations are the suitability for specific drugs only (lipophilic drugs), energy-consuming, time-consuming, and the possible coalescence of particles through the evaporation process [107]. A limited energy-consuming homogenization method (e.g., phase inversion composition (PIC) method) is more preferred for scaling up production [123].

### 5.2. One Step Procedure

In one step procedures the emulsification step is not critical for nanodroplet formation. The one step procedures include: Nanoprecipitation procedure, Dialysis, and superficial fluid technology.

#### 5.2.1. Dialysis

The dialysis method is a one-step method that has been used effectively to produce NPs with narrow size distribution and suitable particle size [124]. The concept of the dialysis method is the reassembly of the polymers due to the reduction of their solubility. Generally, the polymer and the drug are dissolved in a suitable organic solvent, moved to a dialysis membrane, and placed for dialysis in a non-solvent phase [107]. Semipermeable membranes with conventional molecular weight cut-off (MWCO) or dialysis tubes are used as a polymer barrier [111]. The aqueous solvent’s displacement into the membrane changes the polymer-solvent solubility, as the solubility of polymers reduces with the increase of the interfacial tension, which leads to polymers aggregate and development of a nanoparticles suspension [107]. The miscibility between the organic polymer solvent and the dilute dialysis solvent is required. Different parameters can affect the size of particles, such as the type of solvent, the solvent’s viscosity, the miscibility with water, the dialysis MWCO, the polymer concentration, and the rate of solvent mixing [125].

The main limitation of this method is the use of high amount of dialyzing medium, which can stimulate the premature release of the NPs content [107]. In addition, it’s a time-consuming procedure, and faced difficulty in scaling up the production.

#### 5.2.2. Nanoprecipitation Procedures

The Nanoprecipitation method, or solvent replacement, is a one-step method that relies mainly on the interfacial participation of the polymer after replacing the organic solvent from lipophilic solvent to aqueous solvent [107]. The desired drug and polymer are dissolved in a water-miscible solvent with moderate polarity, and then the solution is injected as one shot into a stirred aqueous solution [126]. The polymer-solvent will diffuse quickly and spontaneously to the aqueous solution, and the nanoparticles will be formed [107]. The Marangoni effect was influential for the sequences of the steps [107]. Reducing the interfacial tension between two different phases will spontaneously increase the surface area due to the rapid diffusion, and small droplets of the organic solvent will be created [107].

The diffusing of the solvent out of the nanodroplets stimulates the participation of polymers as nanospheres or nanocapsules. Acetone is one of the commonly used organic solvents in this process due to its miscibility with water and the ease of evaporation [107]. Also, solvent mixtures can be used, such as mixing the methanol or ethanol with a small quantity of water [127]. The protocol of this method is to inject the organic solvent into the aqueous solvent, but the process can be conversed without affecting the formation of the NPs.

Compared to the emulsification solvent-evaporation method, the nano-participation particles are characterized with appropriate particle size and limited size distribution [107]. Regulating the different experimental conditions can impact the final NP properties, such as the ingredient’s nature, ratios of organic solvent to the aqueous solvent, the mixing speed, and the injecting rate [126]. For example, a reported study has shown a variance in the particle size by using different solvents (e.g., acetone, acetonitrile, and tetrahydrofuran), that the most water-miscible solvent (acetone) produces the smallest particles [113]. Chancón et al. [128], investigated the effect of polymer concentration, injection rate and needle gauge, the lowest polymer concentration, lowest needle gauge, and the highest injection rate produced smaller particle sizes (~46 nm) [128].

In general, this method is commonly used due to its rapid, simple, and reproducible results. However, the method is restricted for lipophilic drugs as the loss of the hydrophilic drugs is expected through the diffusion to the aqueous phase [107]. Controlling the quality of the drug, polymer, solvent, and non-solvent system is the nanoprecipitation method’s challenge [126]. Moreover, controlling the mixing process at a constant rate during the nano-precipitation is the main challenge of this method [107].

The microfluidic method could be a promising approach to overcome the uncontrolled mixing process by maintaining the hydrodynamic flow of the solvents at a constant rate and ensuring a well-controlled mixing [129].

## 6. Microfluidics

Microfluidics is a novel, versatile technology that has been developed recently and has been utilized in several areas. A microfluidic system can manipulate a small fluid volume (10^−9^ to 10^−18^ L) using micrometre channels, microvalves, and micromixers as an interconnected system [130]. The principle of microfluidics is based on the rules of a controlled mixing process [131]. By studying the material nature in a number of previous studies, it has been realized that the fluid in microscale has different flow dynamics from those in the macroscale [131]. The flow of the fluid can be categorized into laminar flow and turbulent flow. Turbulent flow is an irregular fluid distribution in different orientations, and most macroscale fluids flow is a turbulent flow. The laminar flow is the parallel orderly flow at constant velocity without scattering of the layers. Laminar flow fluids are controlled by viscous forces rather than inertial forces, which means a constant and continuous flow [132]. The microfluidic system provides microscale continuous laminar flow; this type of flow offers a high mixing quality and enhances the performance of microscale devices [133]. The constant continuous flow provides the same quality of production over time and limiting the batch-to-batch variability [133]. Many studies have described microfluidics as a mechanism to manage the mixing process on a microscale [134,135].

The microfluidic chips have been manufactured by different materials, with monocrystalline silicon and glass borosilicate are the most common due to their suitable properties [131]. However, the disadvantages of these materials, such as the high cost, the brittle nature, and the lack of self-sealing, lead to drawbacks and influence the development of new materials and manufacturing methods [136]. The microfluidic chip’s fabrication material should have appropriate properties, such as flexibility and elasticity [137]. Different polymers (e.g., Polymethyl Methacrylate (PMMA), Polystyrene (PS), and Cyclic Olefin Copolymer (COC)) hold these properties and have been widely used to fabricating microfluidic devices. The polymer structure and manufacturing method can modify the degree of flexibility, rigidity, elasticity, and brittleness. Both, the polymer type and manufacturing methods, can be determined depending on the application of microfluidics [137].

The polymers used for the chip manufacturing can be categorized into four groups of elastomers, thermoplastics, thermosets and, thermoplastics elastomers [137]. The main thermoplastics and elastomers that are used for microfluidic fabrication, include Poly(dimethylsiloxane) (PDMS), Poly (ethylene terephthalate) (PET), polymethylmethacrylate (PMMA), and Cyclo-olefin polymer (COP) (Table 2).

Other thermoplastics that have the potential to be used for chip fabrication include polycarbonate (PC), polystyrene (PS), perylene, and polyvinylchloride (PVC) [144]. In addition, other types of materials could be used to fabricate microfluidic devices such as hydrogels, especially for biological applications (cell-related), and paper, particularly for the open channel microfluidic systems [144]. Several methods have been used to fabricate the polymer’s microfluidic devices, including hot embossing or imprinting, injection molding, laser ablation, soft lithography or X-ray photolithography and 3D printing [145]. 3D printing is a modern technology that has been used recently to fabricate microfluidic devices. Compared to other mentioned microfluidic fabrication systems, 3D printing is low-priced, time-saving, and multiple-purpose [146].

Microfluidics offers several useful applications in different fields, such as electronics, chemistry, biology, medicine, and material science, among many others. The principal innovation of microfluidics is the facility to transfer the traditional bulk technique to 100 nm width microchannels. Within these channels, solvents can be mixed by a pumping system and can be exploited for analysis, separation, and synthesis purposes. For example, microfluidics allows material synthesis, especially nanomaterials and nanostructures, enhancing the production of micro/nano-sized DDSs [147,148]. For example, Wang et al. [149], have used the microfluidic system to create a DDS of exosomes. Exosomes are a novel therapeutic delivery system that suffer from poor targeting ability and lack an effective isolation technique. The workers used a microfluidic system to isolate the exosomes by lining the wall with carbon nanotubes by chemical deposition to catch the exosomes and add the CD63 as a targeting agent to help the exosomes achieve targeted deliver. The system was investigated on cancer cells, and the results showed that exosomes show a significant targeting efficiency with high cellular uptake when exposed to the cells, which enhanced the anticancer effect of the used drug. The results can present that microfluidics was an effective technique to isolate exosomes and improve the targeting capacity [149].

### 6.1. Microfluidics in Nanomedicine

Nanomedicine is one of the most critical topics of present-day investigations. NPs have been approved for clinical use and utilized for multiple medical purposes, including drug and gene delivery. Different types of NPs can be used for the encapsulation of pharmaceutical molecules, including lipid based and polymeric NPs. Several traditional methods have been used for NP manufacturing as discussed previously in Section 4 and Section 5. The utilization of microfluidic system in NP manufacturing will be discussed in this section.

#### 6.1.1. Microfluidics in the Manufacturing of Liposomes

**Figure 4:**
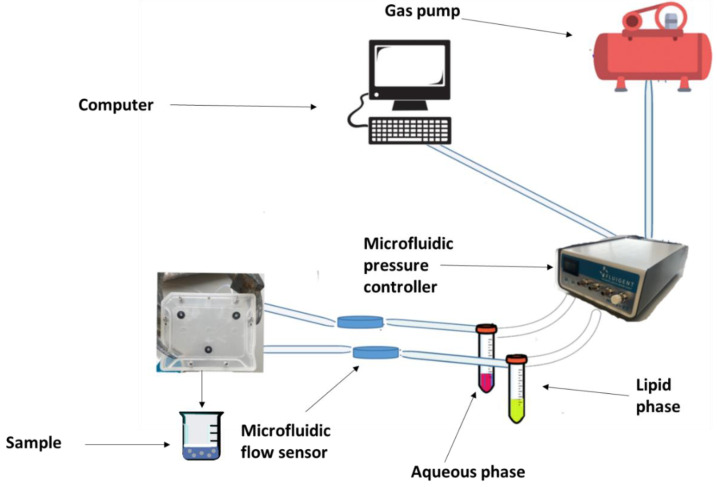
Schematic presentation of liposomes manufacturing using the microfluidic system. Figure adapted from Weaver et al. [152].

As a novel and unique technology, the MF system utilizes microscopic channels that vary in range of dimension (5–500 μm). The power of these channels is in providing the laminar flow of fluids, which enhances the performance and mixing quality of microfluidic devices. In the MF process, the lipids dissolve in a suitable organic solvent (ethanol or isopropanol). The lipid can consist of different types of phospholipids and is usually coupled with cholesterol; the ratio between the lipid and cholesterol highly affects the characteristics of the final formulations. The pre-prepared lipid phase is moved to a specific chamber, and the aqueous phase is transferred to the opposite one (Figure 4). The flow rate ratio (FFR) and total flow ratio (TFR) are adjusted to control the flow of both phases. Then, the mixing process of the organic and aqueous phases is started as a continuous controlled mixing. Mixing the fluids causes a local diffusion of phospholipids in the aqueous phase, which promotes the lipids’ self-assembly and consequently produces liposomes.

MF technology provides a high control on the flow rate, which allows a continuous production of monodisperse and homogenous liposomes. Compared to different MF methods, such as droplets, micro hydrodynamic focusing (MHF), and the pulsed jet flow MF, the MHF method is the most utilized method to fabricate liposomes; furthermore, it has the potential to produce SUVs and LUVs with an appropriate size and homogeneity by a one-step procedure. The success of the MHF method was reported firstly by Jahn et al. [150], reporting the production of liposomes using the perpendicular flow of lipid dissolved in an organic solvent mixed with an aqueous buffer. Several parameters can critically impact the production process, such as the total flow rate (TFR), the flow rate ratio (FRR), the lipid concentration, the solvents, temperature, and the combination of lipids. For example, Aghaei et al. [151] used flow-focusing MFs method, to encapsulate methotrexate within liposomes. This study investigates the impact of different parameters, including TFR, FFR, and total lipid concentration, on the resultant physicochemical characteristics such as particle size, PDI, and encapsulation efficacy of the liposomes. The particle size of the liposomes was within 90–230 nm, and PDI ≤ 0.32 whilst also boasting a high efficiency of encapsulation (≥60%). The drug release studies display a sustained release of the drug and long-acting effect up to 72 h (≤48%). The high encapsulation efficacy and slow drug release directly relate to the manufacturing process [151].

Guimarães et al. [153] have used dimyristoylphosphatidylcholine (DMPC) and distearoylphosphatidylcholine (DSPC) to prepare liposomes using two different methods, the microfluidics and the thin-film hydration method. The results of the different formulations were compared to assess the positive and negative impacts of microfluidics. Comparing the two processes shows that microfluidics is a fast, simple, and single-step technique for liposome manufacturing. The versatility of the method makes the encapsulation process faster with keeping the encapsulation efficiency high. Also, the single-step process of microfluidics makes the scaling up of the production more reliable by reducing the variables experienced within the process. However, producing large industrial patches using microfluidic technology is still a challenge, which is hindering its wide-scale implementation as a formulation method. This is mainly due to the high cost of microfluidic systems, although once they are established, multiple systems running in parallel could provide continuous production of liposomes. Once an established synthesis method is chosen for liposomal production using microfluidics, it could attract various sources of funding which could lead to it eventually being used as a method to provide materials for clinical use.

Gkionisa et al. [154], reported the manufacturing of co-loaded liposomes using thin-film hydration and MFs and comparing the performance of both formulations. Using different methods, DSPC is used for manufacturing liposomes and loading them with doxorubicin: umbelliprenin chemotherapy. The physicochemical properties and toxicity profile compared for both formulations. The study investigated the ability of MFs to provide cost-effective production of co-loaded liposomes with enhanced stability and advanced drug loading capacity. The results show a controlled particle size (100–250 nm), uniform formulation, and doxorubicin drug loading of >80%. Both formulations were examined in a panel of human breast cancer cells to test their potency. MFs shows a quick, reliable method to produce size-controlled liposomes with preserved doxorubicin efficacy and presents low toxicity effect in human breast cells in vitro [154].

The second microfluidic method is the droplet microfluidic process, which requires the dissolution of the phospholipids in hexane solvent to prepare large liposomes (4–20 μm). The use of the organic solvent hexane during the manufacturing process of liposomes can lead to a risk of toxicity. Also, the pulsed jet flow process looks like a modified form of the film hydration method, that the lipid phase should be dried inside microtubes. That is followed by hydration of the resulted lipid film by insertion and perfusion process to create more giant vesicles, 200–534 μm, with an exceptional encapsulation efficiency [155,156]. This method’s primary advantage is given an opportunity to produce vesicles with the wanted size because of the methods’ versatility and flexibility. The main limitations of this method involve the difficulty for large-scale production, as well as the commanding use of organic solvent and extreme agitation [75].

#### 6.1.2. Microfluidics in the Production of SLNs

Solid lipid nanoparticle (SLN) is a promising alternative for the conventional NP-based DDS (e.g., lipid emulsions, polymeric, liposomes). SLN formulation has shown high stability, biocompatibility, and well controlling of the drug release [139]. Despite that, the broad size distribution, the unreproducible formulation and the difficulties in scaling-up lead to multiple drawbacks of SLN formulations [139]. MF has the potential to overcome the limitations of the conventional methods, for example, reducing the consumption of the organic solvents, avoiding the wasting of large quantities of drugs and reagents, and provides a low-cost automated system. Compared to the other conventional method for SLN manufacturing, MFs offers high control on the process parameters and conditions [157]. The MF constant continuous flow with well-controlled conditions produces SLN with controlled particle size, narrow size distribution, high reproducibility, and low batch-to-batch variations [139].

Based on the literature [139,158], most of the papers that used microfluidics for SLN manufacturing use the same procedure. The reported procedure is started by dissolving both lipid and drug in an organic solvent (e.g., ethanol) and inserting thus into the MF device as the inner fluid beside an aqueous phase with a surfactant inserted as the continuous outer fluid. Both fluids could flow at a constant rate from separated syringes to the MF tubes and then to the chip [158]. Regulating the lipid to drug concentration, FRR, and flow velocity could affect the final SLN results. After the SLN is formed, the organic solvent should be evaporated, and the particles cooled at 4 °C. An additional purification step should be performed using a dialysis bag to remove the residual surfactant and the non-encapsulated drug [157].

Arduino et al. [139] have investigated the effect of utilizing the microfluidic system on optimizing the SLN characteristics. SLN formulation, was prepared using the bulk method oil-in-water homogenization process and a MF method to compare the physicochemical characteristics of both formulations. The SLN was also loaded with paclitaxel (PXT) and sorafenib (SFN) to test the anti-proliferative efficiency of SLN produced by MFs and those produced by the bulk method. There was a significant variation in the particle size and PDI of the different formulated SLN. MFs produced SLNs with a small particle size (121 ± 0 nm) and well controlled PDI (0.11 ± 0.20). On the other hand, the bulk method SLN was larger (246 ± 6 nm) and less homogenous (PDI 0.32 ± 080). The encapsulation efficiency of PXT and SFN was more effective in the MF formulation, and SLN-PXT achieved the best encapsulation efficiency (54%). The cytotoxic studies of microfluidic SLN were tested in human alveolar adenocarcinoma (A549) and human glioblastoma cell lines (U87-MG). The results, show that PTX-SLNs and SFN-SLNs provide a potent anti-proliferative effect and higher capacity to penetrate the cancer cells than the free drug [139].

However, research using MFs to produce SLN is still limited. Most of the MF applications are centred on manufacturing liposomes or polymeric NPs. The use of this technology to make SLNs seems to be a promising area of research, especially since it has overcome many limitations of the conventional method. One of the main challenges of manufacturing SLN by MF is concerning the microfluidic chips fabrication material [131].

PDMS based MF chips are sensitive when used with organic solutions, which is a fundamental component of SLN production as well as the lipophilicity of the PDMS that may lead to lipid aggregation or adsorption to the microfluidic chip inner surface [131]. Despite that, using a MF glass or silicon chip can resolve these limitations due to their chemical inertness.

#### 6.1.3. Microfluidics in the Production of Polymeric Formulations

Polymeric NPs gained a high interest in the DDS field, due to their capacity to provide a controlled drug release profile and effectively encapsulate various drugs and adjust their release by altering the use of the polymer characteristics [159]. For example, PCL has a slow degradation rate, making it useful for long-acting formulations [160]. Polymeric NP systems are commonly used to improve the solubility, reduce toxicity, and control the retention time of the drug [161]. The effectiveness of the polymeric NPs is based on the polymer’s nature, the composition of the polymer, and the manufacturing method (discussed in Section 5) of the polymeric NPs [159]. The main limitations of the polymeric NP manufacturing methods include time consumption, their capacity for specific type of drugs (hydrophilic or hydrophobic), requirement for a solvent evaporation step, and demand for a high quantity of the used materials. MFs has been recognized as an advanced method that can overcome some of these limitations and improve the manufacturing of PNP process. MHF is commonly used for the manufacturing of PNP. A suitable polymer can be dissolved in an organic solvent and mixed with an aqueous stream at specific TFR and FFR. The technique of MFs is inducing the lipids self-assembly to produce monodispersed NP without solvent evaporation within seconds [162]. For example, Abstien et al. [163] compared the particles size and polydispersity of two different PNP formulations, one of them is prepared by bulk method (nanoprecipitation) and the other by MFs. A copolymer composed of poly (lactic acid)-poly (ethylene glycol) (PLA-PEG) and poly (lactic-co-glycolic acid) (PLGA) has been used to manufacture PNP. The different parameters of MFs such as TFR, FFR, solvent to aqueous ratio are investigated in this study. The TFR was changed from 2 to 17 mL min^−1^, and the FRR of the aqueous phase to the organic polymer solution was either 10:1 or 5:1. The particle size of the bulk preparation method was larger (52–65 nm) with wide distribution, and the particles prepared by MF produced a smaller particle size (24–43 nm) with the monodispersed distribution. Also, the MFs’ parameters impact on particle size has been investigated and the FFR has been identified as the key parameter to control particle size and improve polydispersity. The results displayed that increasing the FFR of an equal aqueous to organic phase formulation produces a smaller particle size, and the PDI was slightly decreased at a low flow rate of 2 mL min^−1^ and improved at flow rates of 7–17 mL min^−1^ (0.064–0.129) [163].

Hanh T.H. Vu et al. [164] have used PLGA to encapsulate rutin (citrus flavonoid) as a PNP using different methods. One of them is prepared using the bulk method (single emulsion evaporation method), and the other is by microfluidics. The comparative study investigates the impact of using microfluidics on particle size, encapsulation efficiency (EE), and zeta potential. Also, the rutin release was tested in bio relevant media and phosphate buffer (PBS). The reported results show that the microfluidic formulation has a higher encapsulation efficiency (34 ± 2%), smaller particle size (123 ± 4 nm), and faster release than the bulk method (EE 27 ± 1%, size 179 ± 13 nm).

MFs has been used to optimize the loading capacity of PNP. For example, Behnke et al. [165], have investigated different MF parameters including FRR, TFR, initial polymer concentration, the concentration of the PVA as a surfactant, and their effect on the improvement of encapsulation efficiency. By using PLGA to encapsulate an anti-inflammatory drug candidate (dual inhibitor of the 5-lipoxygenase-activating protein (FLAP) and the microsomal prostaglandin E2 synthase-1 (mPGES-1)), which are named BRP-18. Clinical studies reported that the formulation lacks a sufficient loading capacity (LC). To increase the loading capacity and overcome the limitation the manufacturing method is changed from bulk to MFs. The impact of several MF parameters have been investigated, such as the concentration of the PVA as a surfactant, initial polymer concentration, TFR, and FFR. The resultant particles were in the range from 120 to 260 and PDI values from 0.05 to 0.2. A full-encapsulation of the drug was reported with 3% BRP-187 (*w*/*w* PLGA), 0.3% (*w*/*w*) PVA as a surfactant, and a polymer concentration of 25 mg mL^−1^. The highest drug loading (7.3%) was observed for the formulation of PLGA with c = 15 mg mL and 10% BRP-187 (*w*/*w* PLGA) with 0.3% (*w*/*w*) PVA. Changing the manufacturing method from bulk method to microfluidic increase the (LC) significantly and simplified the whole procedure [165].

#### 6.1.4. Lipid–Polymer Hybrid NPs

Lipid–polymer hybrid nanoparticles (LPHNPs) are a new generation of NPs that merge the biocompatibility of lipid polymer and the high stability of polymeric NPs [166]. This hybrid system shows a high encapsulation efficiency, cellular targeting properties, and well-controlled release [166]. Tahira et al. [167], have used MF to encapsulate Sorafenib (chemotherapy) into LPHNPs. The LPHNPs were composed of PLGA, Lecithin, and DSPE-PEG 2000. In the study, the LPHNPs formulation were prepared by a bulk method and MFs, and the encapsulation efficiency results were compared. The formulation produced by MFs shows a higher encapsulation efficiency (95.0%, 93.8%, and 88.7%) and a well-controlled release in comparison to the formulation prepared by the bulk methods. The cell-growth inhibition experiment, safety and stability studies have been performed on breast and prostate cancer cells. The results show that the LPHNPs formulation was more stable, less toxic, and more potent in inhibiting cancer cell growth than free Sorafenib [167].

#### 6.1.5. Microfluidics in Synthesis of Inorganic Nanoparticles

Recently, microfluidics has been used to synthesize different types of inorganic NPs, including gold [168], quantum dots [169], and magnetic NPs [170]. The high controlled system of Microfluidics can produce inorganic NPs with higher quality than those produced with conventional methods. For example, Larrea et al. [170], have used gas slug microfluidics to create a magnetic nanostructure. The results show that microfluidic gas slugs can produce iron oxide nanoparticles with high purity and provide high control on the product characteristics (shape, size, and crystalline phase). As well as increasing the reproducibility of the iron oxide nanoparticles due to the reduction in the total manufacturing process duration.

## 7. Conclusions & Future Predictions

The nanoscale DDSs shows significant success in targeting drug delivery and improving the whole therapeutic process. NPs have been employed in various drug delivery formulations that are today in markets and utilized for disease therapy or diagnosis. The development of drug-loaded NPs from the preclinical stage to the industrial scaling up is challenging due to the difficulty of producing a size-controlled, reproducible, stable formulation with high encapsulation efficiencies and low batch-to-batch variety. The particle size, PDI, and encapsulation efficiency are affected mainly by the manufacturing method. The different NP conventional manufacturing methods are limited in producing NPs with small particle size and narrow size distribution. Also, the multiple steps of the traditional manufacturing procedures (e.g., using mechanical energy for mixing or milling, the requirement for an additional purification step) make them time-consuming, high cost, and negatively affect the properties of the particles. The development of the microfluidic system provides a one-step procedure with a constant and continuous process of NP production. The advanced microfluidic design enables the automatic adjustment of the process parameters, making the process controllable and enhances the possibility to produce the optimum particle size and PDI. Many comparative studies are reported in the literature displaying the difference between the bulk methods and microfluidic method in the assembled particle size, PDI, and encapsulation efficiency. Moreover, cell culture studies and cytotoxicity studies, confirm that enhancing the physicochemical properties of NPs can improve their therapeutic effectiveness, boost potency, and reduce the toxicity of the drug. It can be concluded that the manufacturing method highly affects the resultant particles. Among all conventional methods, the NPs produced by microfluidics methods are more desirable in size, PDI, morphology, therapeutic efficacy, and toxicity.

Future advancements of microfluidic systems can optimize NPs production, extend their use in therapy, and integrate them into new medical applications. Coupling the system with complementary devices (e.g., Process analytical technology (PAT)) can control, analyze, and observe the sample changes during and at different stages of the particle’s fabrication process. Inserting PAT probes can test the physicochemical properties, including the particle size, surface area, surface charge, and morphology beside the FTIR analyses of the manufacturing particle. This significant modification will make the formation, characterization, and testing of NP a complete, interconnected system. Also, incorporating molecular imaging technology (e.g., cryo-EM, XFEL) with the organ-on-chip microfluidic system for addressing the sample delivery shows promising results.

Microfluidics is a high-yielding field for research and development. Improving the microfluidic system itself or merging it with another device to develop NPs, process en-bloc is a vital step for the future of NPs. MFs in the near future can expect innovative devices, solutions, and designs to be applied in the NP field and for different new medical applications.

## Figures and Tables

**Table 1:** Comparison of the Advantages and disadvantages of manufacturing methods of lipid NP and polymeric NP.

Nanoparticles Type	Manufacturing Method	Advantages	Disadvantages	References
Lipid formulation	Film hydration	1- Established method 2- Understood method	1- High consuming of the organic solvents 2- High PDI 3- Lack of reproducibility 4- Need for additional downsizing step 5- Difficulties in scaling-up	[108]
	Solvent injection	1- Simple and fast 2- Scaling- up possibility	1- Exposing to organic solvent 2- high PDI 3- stability problems	[108]
	Extrusion	1- uniform and homogenous formulation	1- possible clogging of the membrane pores. 2- Difficulties in scaling-up	[109]
	High pressure homogenization	1- Scaling- up possibility 2- Uniform formulation	1- High energy consumption 2- Multiple steps 3- Bulky system	[108]
	Microemulsion	1- Small particle size 2- Homogenous formulation	3- Difficulty in removing the excess water 4- Use high concentration of surfactants	[110]
Polymeric	Emulsification -salting out	1- Avoids surfactants and chlorinated solvents.	2- Need for purification steps 3- Encapsulate lipophilic drugs only	[107,111]
	Emulsification- solvent diffusion	1- Scaling- up possibility 2- Batch-to-batch reproducibility	3- The possible diffusion of the hydrophilic drug into the aqueous phase 4- The need to eliminate high volume of aqueous phase from the colloidal dispersion	[107,112]
	Emulsification- evaporation	1- Simple and versatile	2- Risk of nanodroplets coalescence during the evaporation process 3- Time consuming	[111]
	Dialysis	1- Effective and simple method 2- Produce PNP with narrow distribution	1- Time consuming 2- Use of high amount of dialyzing medium, which stimulate the premature release of NPs content	[111,113]
	Nonparticipation	1- simple and established method 2- use low concentrations of surfactant	1- Restricted for lipophilic drugs 2- Low polymer concentration obtained	[107,111]

**Table 2:** Summary of materials used for microfluidic chips, including advantages and disadvantages.

Material	Tg 1 (°C)	Advantages	Disadvantages	Manufacturing Method	References
PDMS (elastomer)	−125	Low Tg, easiness of shaping in the channels, optical transparency, resistance to water, ability to produce microscale features precisely.	hydrophobic nature, sensitive to organic solvents (e.g., strong acids, hydrocarbon, amines.	soft lithography, plasma-enhanced bonding	[137,138,139]
PMMA	105	low cost, optical transparency, attractive mechanical/chemical characteristics, and simple fabrication processes.	High Tg, Sensitive to alcohol, isopropyl alcohol and acetone, high bonding temperature, Commercial availability	solvent imprinting, hot embossing thermal bonding, injection molding and laser ablation.	[140,141]
PET	69–78	Low Tg, low rigidity, low surface energy, easiness of molding, chemically inertness, good gas and moisture barrier characteristics, recyclable.	reduced chemical resistance, require surface treatment for bonding due to the low plasma bonding strength.	hot embossing, thermal bonding	[137,142]
COP	70–180	High stability, low interaction with protein, suitable rigidity, resistance to almost all solvents including ethanol, IPA, and acetone, low water absorbency, high moisturiser barrier.	High Tg, brittleness and low heat diffusivity, not resistance to the non-polar organic solvent (e.g., hexane).	hot embossing, chemical etching, thermal bonding methods.	[143]

## Data Availability

No new data were created or analyzed in this study. Data sharing is not applicable to this article.
